# Acute Glycemic Control in Prediabetes Individuals Favorably Alters Serum NLRP3 Inflammasome and Related Interleukins

**DOI:** 10.3390/ijms241813837

**Published:** 2023-09-08

**Authors:** Hend Alfadul, Shaun Sabico, Abdullah M. Alnaami, Osama E. Amer, Syed D. Hussain, Kaiser Wani, Mario Clerici, Nasser M. Al-Daghri

**Affiliations:** 1 Chair for Biomarkers of Chronic Diseases, Biochemistry Department, College of Science, King Saud University, Riyadh 11451, Saudi Arabia; 2 Department of Medical-Surgery Physiopathology and Transplantation, University of Milan, 20122 Milan, Italy

**Keywords:** prediabetes, type 2 diabetes mellitus, NLRP3, inflammasomes, interleukins, chronic inflammation, inflammatory disease

## Abstract

Hyperglycemia associated with prediabetes (PD) alters NLRP3 inflammasome activity and related interleukins, yet no study has evaluated the expression of the NLRP3 inflammasome complex and related interleukins in individuals with a PD condition that did or did not develop type 2 diabetes mellitus (T2DM). This study investigated the effect of 6 months of lifestyle modification on the expression of the NLRP3 inflammasome and related interleukins (1α, 1β, 18, 33 and 37) in the sera of individuals with a PD condition that did or did not develop T2DM. This interventional study included 67 Saudi adults (mean age = 41.9 ± 8.0 years, mean BMI = 33.2 ± 5.5 kg/m^2^). Overnight-fasting serum samples were collected at baseline and at the 6-month follow-up. Serum levels of NLRP3, capsase-1 and related ILs were analyzed at both visits using commercially available immunoassay kits. Results showed that IL-1α increased in the PD group that developed T2DM (*p* = 0.046), IL-33 decreased in the PD group that reverted to normal (*p* < 0.001) and NLRP3 decreased in the PD group that remained PD (*p* = 0.01). Results also showed a positive over-time correlation between NLRP3 and both IL-1α and IL-33 (*p* < 0.001 and *p* = 0.028, respectively). In conclusion, glycemic control favorably altered NLRP3 inflammasome complex activity, and lifestyle modification in PD individuals is crucial in reversing harmful metabolic and inflammatory phenotypes.

## 1. Introduction

Global evidence indicates an increase in diabetes mellitus (DM) with no signs of a halt any time soon [1]. The World Health Organization reported an 80% increase in DM in the 34 years between 1980 and 2014 [2]. Data published in 2019 from the International Diabetes Federation (IDF) showed that around 9% of adults around the world live with DM, and around 7% (3.7 × 10^8^) live with prediabetes (PD), which is predicted to rise to around 8.5% (5.4 × 10^8^) by the year 2045. The Middle East and North African (MENA) region held the highest incidence of DM at around 12% [1]. In the Middle East, the Kingdom of Saudi Arabia (KSA) in particular has the second highest incidence of diabetes; around 24% (7 × 10^6^) of Saudi adults have type 2 diabetes mellitus (T2DM) and 10.3% (3 × 10^6^) have PD [3]. Because of this increased prevalence of PD in the KSA, the burden of DM is sure to rise [1]. 

PD is an intermediate dysglycemic state that resides between euglycemia and T2DM. The pathogenesis of dysglycemia has been elucidated thoroughly. It involves two stages in the asymptomatic phase: the first one is the PD stage, which lasts roughly 8–10 years, and the second one is the preclinical diabetic stage, which lasts roughly 4–7 years [4]. In PD, blood glucose levels are higher than the normal range but not high enough to fulfill the diagnostic criteria for T2DM [3,5]. The diagnostic criteria for PD according to the American Diabetes Association (ADA) include having one of the following three conditions: 1. hemoglobin A1 concentration (HbA1c) levels between 5.7 and 6.4%; 2. fasting plasma glucose (FPG) levels between 100 and 125 mg/dL; 3. two-hour oral glucose tolerance test (2h-OGTT) levels between 140 and 199 mg/dL [6].

Managing and reversing PD is very important in preventing T2DM and its related complications, as there is a 5–10% risk of developing T2DM within 12 months for individuals with unmanaged PD [7]. The development of T2DM from PD progresses gradually over time, and it was thought that once it happens, it is irreversible [3]. Conversely, emerging reports are now challenging this belief, with new tactics to control and reverse T2DM [8,9]. According to Diabetes UK, this can be achieved via exercise, diet restriction and weight-loss surgery [9]. For this reason, a thorough understanding of the PD state and direct intervention is crucial in delaying or even better preventing the development of T2DM [3]. PD is not only associated with the development of the full-blown diabetic disease down the line but is also associated with heart disease and a poor prognosis [1,5]. PD individuals have an increased risk of cardiovascular disease and other chronic conditions [5,7]. A large meta-analysis published in 2016 that aimed to observe the prognosis involved with PD and compare it to healthy controls showed that PD was associated with higher risks of cardiovascular disease and death [10]. Some of the significant risk factors associated with PD include age and body mass index (BMI); results have shown a significant age-correlated rise in PD. The presence of PD is drastically greater in obese people compared to non-obese people. Eight out of ten individuals with PD are obese or overweight. The increase in both PD and obesity around the world is indeed a massive issue [11]. Because PD is an asymptomatic disease, the ADA advises screening for PD in adults with BMIs equal to or greater than 25 Kg/m^2^ [3]. Obesity induces inflammation, which causes diseases with glucose and lipid metabolism dysregulation, like coronary heart disease, insulin resistance and DM [12,13].

Hyperglycemia associated with PD results in increases in oxidative stress and pro-inflammatory biomarkers, which eventually lead to coronary heart disease [14,15,16]. Research has proven that, in addition to pro-inflammatory markers, anti-inflammatory biomarkers are also increased in disease development [17]. Different pro- and anti-inflammatory interleukins (ILs) from the IL 1 family were correlated with the development of T2DM from PD (e.g., IL-18 [14] and interleukin 1 receptor antagonist (IL-1RA) protein [15]). In PD individuals, the IL-1RA levels were higher than the IL-18 levels [15]. A possible explanation for this is that the body is trying to counterbalance the high pro-inflammatory environment. Still, the anti-inflammatory response seems to be insignificant, as the increase is incompetent at halting disease development [18]. While the glycemic status changes from euglycemia to PD to the full-blown T2DM disease, the inflammatory biomarker profile fluctuates [15]. This fluctuation is extremely critical, as it permits the distinction between the asymptomatic preclinical and clinical stages of this disease [19]. Glycemic dysregulation activates different pathways that all ultimately stimulate the nuclear factor kappa-light-chain-enhancer of activated B cells (NF-κB) signaling pathway [19]. For instance, high mobility group box 1 (HMGB1) was increased in macrophages exposed to high glucose, which caused the hyper-activation of the NF-κB and mitogen-activated protein kinase (MAPK) signaling pathways [20].

The activation of the innate immune system and the resulting chronic inflammation play an important role in the development of DM [21]. The innate immune system is activated via pattern recognition receptors (PRRs). PRRs detect pathogen-associated molecular patterns (PAMPs) or damage-associated molecular patterns (DAMPs). Nucleotide-binding oligomerization domain like receptor protein 3 (NLRP3) is a vital cytosolic PRR. First discovered in 2002 by Martinon and colleagues [22], NLRP3 is the most analyzed inflammasome complex. It is a tri-protein complex that consists of the NLRP3 sensor protein, the apoptosis-associated speck-like protein containing a caspase recruitment domain (ASC) linker protein and the precursor caspase 1 effector enzyme (pro-caspase1) [23]. The NLRP3 inflammasome requires two signals for its activity: a priming signal and an activation signal [24]. In the priming signal, DAMPs or PAMPs trigger IL-1β receptors (IL-1Rs), Toll-like receptors (TLRs), or tumor necrosis factor receptors (TNFRs), which then triggers nuclear factor kappa B (NF-κB) translocation to the nucleus, which stimulates the transcription of NLRP3 and IL-1β. In the activation signal, various cellular and molecular measures have been suggested as the triggers for NLRP3 inflammasome oligomerization and activation. Active caspase-1 produced from NLRP3 inflammasome activation cleaves and activates precursor interleukin-1β (pro-IL-1β) and interleukin-18 (pro-IL-18) into their mature biological forms, IL-1β and IL-18, respectively [13]. To sustain a reduced inflammatory environment, NLRP3 inflammasome activity is maintained low under regular physiological states [24]. During the last decade, it has been well documented that dysregulation in NLRP3 inflammasome activity is involved in diabetes development [24]. Even though the significance of NLRP3 inflammasome hyper-activation in T2DM progression is well documented in both humans and animals, the activity of the NLRP3 inflammasome in PD individuals remains a gray area that has not been well investigated until now [25]. It is hypothesized that the status of NLRP3 activation can be identified as surrogate biomarkers that allows for the prediction of which individuals with a PD condition will progress to develop the full-blown disease.

As previously mentioned, PD is a strong risk factor for T2DM and is strongly associated with heart disease, and early intervention is vital [26]. Lifestyle changes are the early initial treatment approach for PD [5]. This was specified by the ADA, who mentioned that the most proficient intervention for PD is lifestyle changes, such as adapting a healthy diet, exercising and having a reduced BMI. Medication-based treatments can be considered in decreasing the risk of developing T2DM. However, they are less efficient than intensive lifestyle-change remedies [27]. This was shown in a systematic review and meta-analysis published in 2018 by Glechner and his team, which showed that after one and three years of lifestyle changes in PD individuals, there was around a 35–55% decrease in the chance of developing T2DM compared to PD individuals who received medication [28]. Prior to this, in a meta-analysis, Glechner and his team also showed that lifestyle changes in PD individuals lowered their risk for developing T2DM, but these results were gender-specific [29]. In almost all major studies that evaluated the effect of lifestyle changes in diabetes development, the effect of these changes continued years after terminating the trials. This effect continuation was the consequence of metabolic memory. Certainly, accumulating results show that interventions to reach euglycemia at earlier stages of disease have significant and longer-lasting outcomes compared to at later stages of the disease [5,30]. The full mechanisms underlying this phenomenon are not yet fully understood.

It is hypothesized that the status of the NLRP3 inflammasome and related interleukins (1α, 1β, 18, 33 and 37) can be identified as surrogate biomarkers that allow for the prediction of PD individuals who will progress to T2DM and/or revert to a normoglycemic status. Thus, in the present study, we aimed to evaluate, for the first time, the longitudinal expression of the NLRP3 inflammasome and related ILs (1α, 1β, 18, 33 and 37) in PD individuals that did or did not develop T2DM, and we found that glycemic control favorably altered the NLRP3 inflammasome complex (NLRP3, IL-1α and IL-33 levels). We also aimed to evaluate the overtime correlation between NLRP3 and the mentioned ILs.

## 2. Results

Table 1 summarizes the general characteristics of the participants at baseline and after a 6-month follow-up according to the following: (A) FSG and (B) HbA1c levels. Based on the FSG levels, after the 6-month follow-up, 13 participants reverted to normal (HC), 48 participants remained PD and 6 participants developed T2DM. There was a significant decrease in the insulin levels in the PD participants that remained PD after the 6-month follow-up (baseline: 16.2 ± 5.1, 6-month follow-up: 15.6 ± 5.0; *p* = 0.005). There was also a significant increase in the triglyceride levels in the PD participants that developed T2DM after the 6-month follow-up (baseline: 1.0 (0.8–1.6), 6-month follow-up: 1.4 (1.0–2.0); *p* = 0.046). There were no significant differences in any other parameters in the PD participants after the 6-month follow-up. Based on the HbA1c levels, after the 6-month follow-up, 31 participants reverted to normal (HC), 28 participants remained PD and 8 participants developed T2DM. There were significant decreases in the weight and BMI in the PD participants that reverted to normal (HC) after the 6-month follow-up (baseline: 81.3 ± 15.9, 6-month follow-up: 80.0 ± 16.4; *p* = 0.02 and baseline: 32.8 ± 6.6, 6-month follow-up: 32.3 ± 6.6; *p* = 0.02, respectively). There was also a significant decrease in the insulin levels in the participants that remained PD after the 6-month follow-up (baseline: 16.3 ± 5.9, 6-month follow-up: 15.8 ± 5.8; *p* = 0.004). There were no significant differences in any other parameters in the PD participants post-intervention.

Table 2 summarizes the circulating levels of NLRP3 and related ILs (1α, 1β, 18, 33 and 37) of PD participants at baseline and after the 6-month follow-up according to the A. FSG and B. HbA1c levels. Based on the FSG levels, the IL-33 levels significantly decreased in the PD group that reverted to normal after 6 months of intervention (baseline: 3.2 (1.1–4.1), follow-up: 0.7 (0.6–1.2); *p* = 0.007) and they significantly decreased in the PD group that remained PD after 6 months of intervention (baseline: 3.3 (2.9–3.9), follow-up: 1.0 (0.6–3.0); *p* < 0.001)). The IL-37 levels significantly decreased in the PD group that remained PD after 6 months of intervention (baseline: 4.2 (2.1–10.7), follow-up: 2.9 (2.1–2.9); *p* < 0.001). Finally, the NLRP3 levels significantly decreased in the PD group that remained PD after 6 months of intervention (baseline: 0.13 (0.1–0.22), follow-up: 0.11 (0.07–0.18); *p* = 0.01). Based on the HbA1c levels, the IL-1α levels significantly increased in the PD group that developed T2DM after 6 months of intervention (baseline: 0.6 (0.5–0.8), follow-up: 1.0 (0.9–1.4); *p* = 0.046). The IL-33 levels significantly decreased in the PD group that reverted to normal after 6 months of intervention (baseline: 3.2 (0.7–4.0), follow-up: 0.8 (0.6–2.1); *p* < 0.001) and they significantly decreased in the PD group that remained PD after 6 months of intervention (baseline: 3.3 (3.0–4.0), follow-up: 1.0 (0.6–2.1); *p* < 0.001).

**Figure 1:**
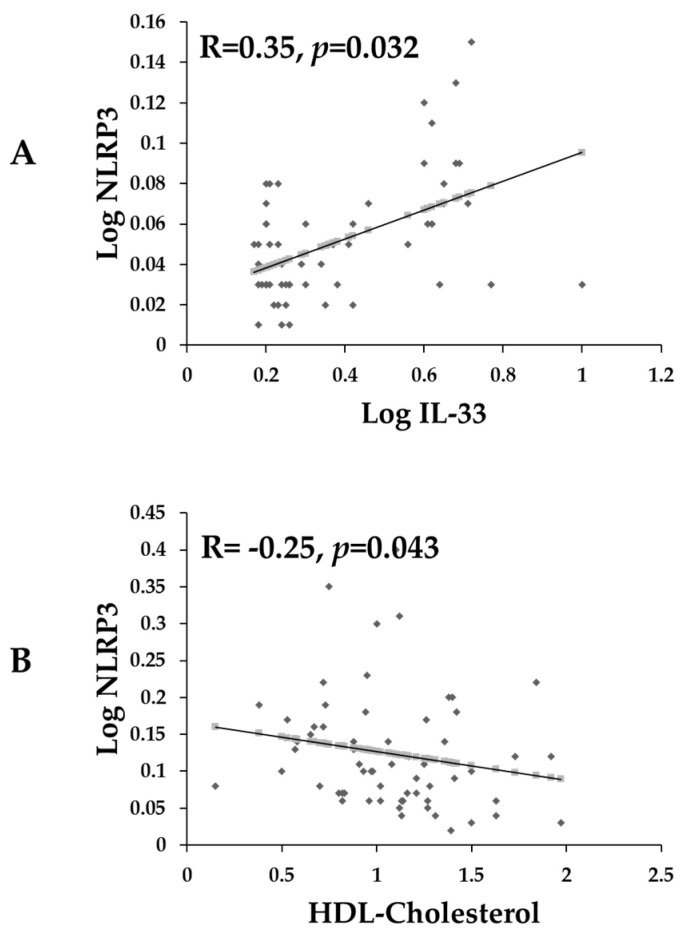
Scatterplots showing the bivariate correlations of NLRP3 with (**A**) IL-33 and (**B**) HDL cholesterol in all pre-DM participants at baseline. Variables were log-transformed before the correlation analysis was performed. A *p* < 0.05 was considered significant.

In Figure 1A, there was a significant and positive association between log NLRP3 and log IL-33 (R = 0.35, *p* = 0.03). In Figure 1B, there was a significant inverse association between log NLRP3 and HDL cholesterol (R = −0.25, *p* = 0.04).

**Figure 2:**
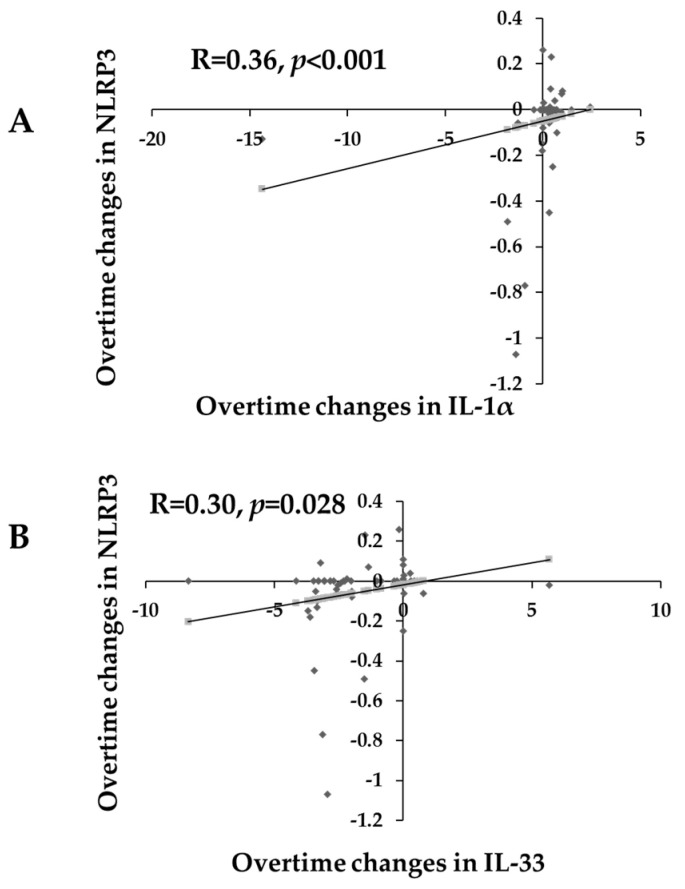
Scatterplots showing the bivariate correlations over time of NLRP3 with (**A**) IL-1α and (**B**) IL-33 in all PD participants.

In Figure 2A, there was a significant and positive association between NLRP3 and IL-1α over time (R = 0.3, *p* = 0.001). In Figure 2B, there was a significant positive association between log NLRP3 and IL-33 over time (R = 0.03, *p* = 0.028).

**Figure 3:**
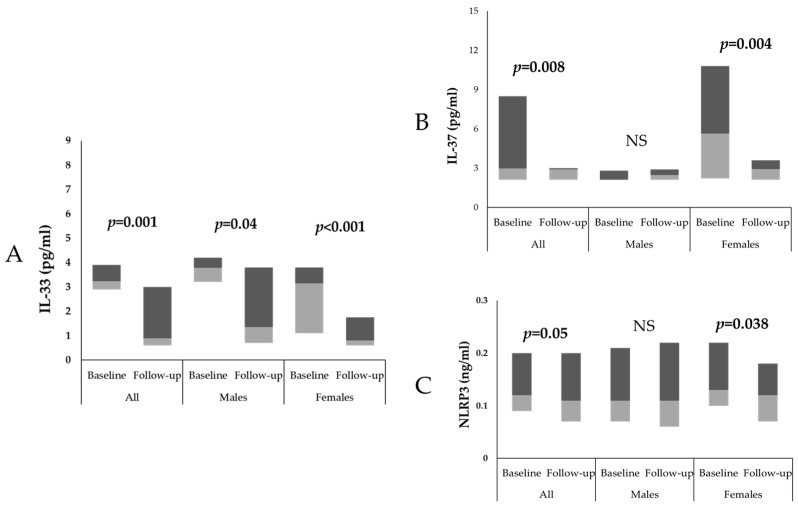
Boxplots showing circulating levels of (**A**) IL-33, (**B**) IL-37 and (**C**) NLRP3 in all participants, in males and in females based on FSG at baseline and at 6-month follow-up. The light-gray and dark-gray boxes show the third and first quartiles, respectively, and the meeting point signifies the median; NS—not significant.

Based on the FSG levels, Figure 3A shows that the IL-33 levels significantly decreased after 6 months of intervention in all participants (baseline: 3.2 (2.9–3.9), follow-up: 0.9 (0.6–3.0); *p* = 0.001), in males (baseline: 3.8 (3.2–4.2), follow-up: 1.4 (0.7–3.8); *p* = 0.04) and in females (baseline: 3.2 (1.1–3.8), follow-up: 0.8 (0.6–1.8); *p* = 0.001). Figure 3B shows that the IL-37 levels significantly decreased after 6 months of intervention in all participants (baseline: 3.0 (2.1–8.5), follow-up: 2.9 (2.1–3.0); *p* = 0.008) and in females (baseline: 5.6 (2.2–10.8), follow-up: 2.9 (2.1–3.6); *p* = 0.004). Figure 3C shows that the NLRP3 levels significantly decreased after 6 months of intervention in all participants (baseline: 0.1 (0.1–0.2), follow-up: 0.1 (0.1–0.2); *p* = 0.05) and in females (baseline: 0.1 (0.1–0.2), follow-up: 0.1 (0.1–0.2); *p* = 0.038).

**Figure 4:**
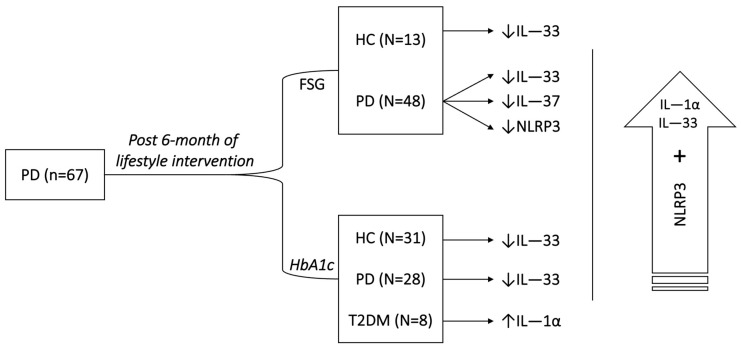
Schematic diagram of major results that indicate the model. ↑ denotes increase in levels over-time while ↓ denotes decrease in levels over-time.

Figure 4 summarizes the major results that indicate the model; the IL-1α levels significantly increased in the PD group that developed T2DM after 6 months of intervention. The IL-33 levels significantly decreased in the PD group that reverted to normal and in the group that remained PD after 6 months of intervention. The IL-37 levels significantly decreased in the PD group that remained PD after 6 months of intervention. The NLRP3 levels significantly decreased in the PD group that remained PD after 6 months of intervention. In addition, NLRP3 showed a positive correlation with both IL-1α and IL-33 over time.

## 3. Discussion

The current study aimed to evaluate the expression of the NLRP3 inflammasome and related ILs (1α, 1β, 18, 33 and 37) in the sera of PD participants who, after 6 months of lifestyle modification, either reverted to normal, remained PD or developed T2DM. It also aimed to evaluate the over-time correlation between NLRP3 and the mentioned interleukins. It is noteworthy that this is the first longitudinal study to evaluate the effect of lifestyle modification on NLRP3 inflammasome activity and related interleukins in association with glycemic control in PD individuals.

Surprisingly, the results indicated that neither the NLRP3 levels nor caspase-1 activity were affected by lifestyle modification in participants who reverted to normal or developed T2DM. However, the IL-1α levels significantly increased in participants who developed T2DM. This is consistent with our recent cross-sectional study, in which we found that the circulatory IL-1α levels were drastically influenced by T2DM [31]. Legiawati et al. found that the IL-1α levels were higher in T2DM patients with HbA1c levels above 7% [32], while Khalil et al. recorded higher serum IL-1α levels in T2DM patients infected with the hepatitis C virus [33]. Mirhafez et al. found that individuals with metabolic syndrome (MetS) had higher serum IL-1α levels than healthy controls [34]. On the contrary, Cantuaria et al. found that the IL-1α levels decreased after high glucose and lipopolysaccharide (LPS) stimulation [35]. The use of an in vitro cell line may be the root cause of this contradiction.

Likewise, the IL-33 levels significantly decreased in the PD participants that reverted to normal after 6 months of lifestyle modification. In PD, it is extremely important that one sees the IL-33 levels increasing, as the immune system is trying to shut down inflammation to impede the development of T2DM. Thus, we believe that IL-33 exerts pro-inflammatory properties, and its decline after lifestyle modification hints at a decline in the inflammatory status. Likewise, Stankovic et al. recorded lower serum IL-33 levels in healthy controls compared to carotid artery disease patients, in which around half of these patients had T2DM [36]. Fan et al. and colleagues analyzed soluble IL-33 in pregnant women with gestational diabetes mellitus (GDM) and normal glucose tolerance (NGT), and they recorded significantly lower levels in the NGT pregnant women [37]. In contrast, Hasan et al. recorded no differences in the adipose tissue IL-33 gene expression amongst normal, PD and T2DM patients [38]. Serum protein levels were not analyzed, making it difficult to compare findings.

Chronic inflammation is a signature of pathogenic development in diabetes. It stimulates both innate and adaptive immune responses, which, in turn, stimulate specialized leukocytes and various interleukin production. Increased calory intake and obesity is an intoxicating trigger to inflammation. Studies have shown that, in addition to aiding in losing weight, lower calory intake and intermittent fasting significantly reduce obesity-associated inflammation [39]. NLRP3 inflammasomes are activated by DAMPs and PAMPs, which increase in an obese state (e.g., glucose, free fatty acids and lipopolysaccharides). This results in the production of various interleukins and chemokines [13,40]. 

The activation of the NLRP3 inflammasome is an important mechanism in lipocyte differentiation and appears to trigger insulin resistance in lipocytes. Lifestyle modification aided in weight loss in obese, T2DM patients and decreased the NLRP3 and IL-1β expression and thus, in turn, the insulin resistance [39]. An ever-increasing number of studies have linked DAMP signals to NLRP3-associated diseases. However, it has been proven that countless NLRP3-associated diseases are linked to a mutation in the NLRP3 gene, and this mutation results in making the protein constantly active even when lacking a DAMP or PAMP signal [41]. In spite of the known direct association between glycemic and macrophage dysregulation, until now, this association has not been fully elucidated, nor is there a treatment plan to control the dysregulated immunity correlated with diabetes side effects [35]. Thus, more research is needed to clarify and explain the effect of glycemic dysregulation on the immune response.

In the present study, the NLRP3 levels were not affected in participants who reverted to normal or developed PD. However, they significantly decreased in participants who remained PD post-intervention. The results also showed that the NLRP3 levels significantly decreased after 6 months of lifestyle modification in all participants. These findings may be the result of adopting a healthy lifestyle through diet and exercise. Conforming to this hypothesis, the current longitudinal study found an inverse association between HDL cholesterol levels and NLRP3. This is consistent with our previous findings [42]. These results are of great significance, as obesity is an important contributing factor in diabetes and this could be seen in this study, in which the weight of the PD group that developed T2DM was already higher than those of the PD group that reverted to normal and the group that remained PD. Circulatory NLRP3 is a potential inflammatory biomarker in various inflammatory diseases [31,43]. These results support our recent cross-sectional study [31], in which we aimed to evaluate the differences in NLRP3 inflammasomes and related ILs in a cohort of PD and T2DM participants. We found higher NLRP3 levels only in the female PD group. That being said, the results still need to be taken cautiously, as the general linear model results indicated that the gender factor was not significant in predicating NLRP3 levels; however, T2DM status, age, IL-18, IL-1α and 33 did [31]. To date, no studies have investigated the effect of lifestyle modification and glycemic control on serum NLRP3 levels.

IL-1α, a pro-inflammatory cytokine, is constitutively expressed and primarily only passively released by pyroptotic cells. IL-1α plays an alarmin role to warn the immune system and activate a sterile immune response [44]. However, a little bit over a decade ago, Gross et al. and Fettelschoss et al. proved inflammasome-regulated active IL-1α release upon Toll-like receptor (TLR) activation [45,46,47]. The release of IL-1α depends on the presence of IL-1β. IL-1β acts as a cargo and binds IL-1α directly, and they are then co-secreted together upon stimulation [47]. In a catalytic-independent way, the presence of caspase-1 is crucial for IL-1α release upon NLRP3 inflammasome activation, feasibly regulating the active release of IL-1α. In our recent cross-sectional study, we showed that NLRP3 significantly predicted IL-1α levels by as much as 43% of the variance, while IL-1α significantly predicted NLRP3 levels by as much as 46% [31]. However, Yazdi and Drexler concluded that IL-1α seems to be more essential in pro-inflammatory responses than the NLRP3 protein, suggesting an alternative pathway that may influence IL-1α release [47]. Further studies are needed to elucidate the role of NLRP3 in IL-1α regulation.

In the current longitudinal study, the IL-1α levels significantly increased in participants who developed T2DM and the over-time NLRP3 levels were positively associated with IL-1α. Mirhafez et al. found that IL-1α was the most potent predictor of MetS, a disease that is linked to a pro-inflammatory milieu [34]. Khalil et al. found that the serum IL-1α levels in hepatitis C T2DM patients correlated with their fasting glucose. They also indicated that circulatory IL-1α can be used as a predictive biomarker for disease pathogenesis [33]. Salti et al. showed that high glucose increased IL-1α production in diabetic nephropathy [48], while Schunk et al. showed that the IL-1α expression was increased on the cell surfaces of leukocytes from acute myocardial infarction and chronic kidney disease patients [49].

IL-33, a new member of the IL-1 family, is involved in the secondary innate immune response. IL-33 is released into the circulation following cell death, warning the immune system of internal damage. Contingent on the environment, IL-33 functions as a double-edged sword that plays both anti- and pro- inflammatory roles [31]. The latest data indicate that serum IL-33 may have protective metabolic outcomes on HbA1c, BMI and lipids, particularly in euglycemic non-obese people. However, this is not the case in obese T2DM patients [38]. IL-33 plays protective roles via various mechanisms: it inhibits resistin synthesis and thus inhibits insulin resistance and T2DM development. It also increases the number of T helper type 2 (Th2) cells, thereby inducing the Th2 immune response and cytokine production, and activating the secondary adaptive immune response. IL-33 also pushes macrophages towards a protective anti-inflammatory M2 phenotype, stimulating cell proliferation and tissue repair [50]. Nuclear IL-33 regulates the gene expression of pro-inflammatory genes via the dampening of NF-κB signaling. In response to DAMPs and PAMPs, IL-33 is released from pyroptotic cells. Acting as a DAMP itself, IL-33 triggers an inflammatory response and, hence, showcases its dual function [51].

The present findings showed that the IL-33 levels significantly decreased in PD participants whose HbA1c and FSG levels reverted to normal after 6 months of lifestyle modification. It also showed the presence of a positive correlation over time between NLRP3 and IL-33. After 6 months of lifestyle modification, the IL-33 levels significantly decreased in all participants, in the male participants and in the female participants. Consistent with this, in our recent cross-sectional study, we showed that IL-33 was positively correlated with NLRP3. We also showed that the NLRP3 levels were significantly influenced by IL-33 and, vice versa, the IL-33 levels were significantly influenced by NLRP3 [31]. In contrast, Hasan et al. found that IL-33 was inversely correlated with the HbA1c in normoglycemic and T2DM participants and positively associated in PD participants. The FSG was inversely correlated in T2DM patients who had better glycemic control [38]. The anti- and pro- inflammatory roles of IL-33 may explain the inconsistency in the results, and further research is needed to elucidate the role and association of IL-33 in meta-inflammatory diseases.

All things considered, our findings indicate that healthy lifestyle modification through diet and exercise improves glycemic control and the inflammatory status and, thus, the PD prognosis. Inflammasome markers can be favorably reversed through lifestyle modification. We faced a couple of limitations: 1. The sample size was small, as participants failed to follow through. 2. The activity of the NLRP3 inflammasome pathway was not analyzed to exclude the influence of other pathways on the analyzed interleukins. 3. The gene expression of the NLRP3 inflammasome pathway and analyzed interleukins was not measured. 4. The serum levels of IL-18 and IL-1β were analyzed using a multiplex immunoassay, which gave unexpected results; there is high heterogeneity even between single-plex immunoassays. Caution is advised in the interpretation of the results [52,53]. The results were inconsistent and thus not taken into consideration. 5. The duration of the study was short; longer studies are needed in which patients are followed up every three months. 6. The activity of non-canonical inflammasomes via caspase-4 and caspase-5 was not analyzed to evaluate the possible involvement of non-canonical inflammasomes on the IL levels. Nonetheless, the current longitudinal study is the first of its kind to elicit the effect of glycemic control on the NLRP3 inflammasome pathway and related interleukins in a PD cohort.

In conclusion, glycemic control modulates the activity of the NLRP3 inflammasome and thus can improve and reverse metabolic and inflammatory phenotypes. Longer and larger-scale studies are needed to confirm whether glycemic control further alters NLRP3 and other emerging regulators of the innate immune system.

## 4. Materials and Methods

The current 6-month lifestyle intervention study took advantage of the fact that King Saud University (KSU) has stored, over time, biological (serum) samples from PD patients who were at risk of developing the disease (T2DM), and who, over time, did or did not progress with clinically evident T2DM. This interventional study was conducted by the Chair for Biomarkers of Chronic Diseases (CBCD), College of Science at KSU, Riyadh, KSA, in cooperation with the diabetes center at King Salman Hospital (KSH) from April 2014 to March 2017. The protocol was approved by the Ethics Committee of the College of Science, KSU (Ref 21/0412/IRB), and was conducted in accordance with the ethical standards set by the Helsinki Declaration of 1975.

### 4.1. Study Design and Participants

This interventional study included 67 Saudi adults (mean age = 41.9 ± 8.0 years, mean BMI = 33.2 ± 5.5 kg/m^2^) attending KSH and other care centers for a regular overnight-fasting blood withdrawal. Consenting patients were registered in the interventional study if they had impaired glucose tolerance (from 5.6 to 6.9 mmol/L was regarded as PD) [54]. Exclusion criteria included pregnant women, emigrants, patients who already had type 1 diabetes (T1DM) or T2DM, patients on diabetes medication, and patients with chronic diseases, like cardiac, renal or hepatic complications. Out of the 138 participants recruited, 67 (20 males/47 females) participants were included in this study. Longitudinal data were taken from the prediabetes project database of the CBCD [7,55,56]. For the purpose of the present study, data from prediabetes participants at baseline and after 6 months were considered. In brief, PD adult participants with no other health complications were lectured by a physician and a dietician on the risk factors and complications associated with PD, and they were also given guiding healthy lifestyle brochures.

Supplementary Table S1 summarizes the baseline general characteristics and circulating levels of NLRP3 inflammasomes and related interleukins of the study participants. A total of 67 (males: 20/females: 47) Saudi adults with prediabetes completed the 6-month follow-up of this intervention. Non-adherence and loss to follow-up were the most common reasons for participants to be excluded from the study.

### 4.2. Intervention

Details of the intervention were published previously [7,55,56]. In brief, a lifestyle modification program aimed at diabetes prevention and targeting the adult prediabetes population in Riyadh, Saudi Arabia, was launched by the CBCD at KSU in collaboration with the diabetes center at KSH. Participants were given a series of lectures twice a month for 3 months covering the program goals, including diabetes knowledge and risk factors, weight management, carbohydrate awareness and physical activity, to name a few. They were also given booklets with beneficial information summarizing the educational sessions with monitoring and checklists. Furthermore, workshops and seminars on related topics were given by researchers three times a year to reinforce the program goals. Participants were also asked to record their caloric intake and physical activity, and they were monitored every 3 months by assessing their anthropometrics and serum biochemical profiles [7,55,56].

### 4.3. Sample Collection and Anthropometrics

Serum samples from the chosen patients were taken from the biobank database of the CBCD, College of Science at KSU, Riyadh, KSA. In short, overnight-fasting whole-blood samples were withdrawn by trained technicians and nurses from all patients. To attain serum from whole blood, the blood was allowed to clot unbothered in red-top tubes on the bench at room temperature for 30–60 min, and the clotted blood was centrifuged in a refrigerated centrifuge (4 °C) for 10 min at 1250× *g* to eliminate the clot. Post-centrifugation, the attained serum was immediately aliquoted, labeled and taken to the CBCD at KSU for abrupt storing in a (−80 °C) freezer until further enquiry. The anthropometrics, which comprised height (cm), weight (kg), waist and hip circumference (cm) and blood pressure (mmHg) evaluations, were taken by trained technicians and nurses. The body mass index (BMI) (kg/m^2^) and waist–hip ratio (WHR) were attained using the following equations: weight in kilograms divided by the square of height in meters for the BMI, and quotient between waist and hip circumferences for waist–hip ratio (WHR). DM status was defined by analyzing FSG and HbA1c; the former reflects acute glycemic control, and the latter reflects long-term control. According to the ADA criteria, an FSG lower than 5.55 mmol/L was regarded as normal, an FSG between 5.55 and 6.9 mmol/L was regarded as PD and an FSG greater than 6.9 mmol/L was regarded as T2DM. HbA1c less than 5.7% was regarded as normal, HbA1C from 5.7% to 6.4% was regarded as PD and HbA1c 6.5 or higher was regarded as T2DM [54].

### 4.4. Biochemical Estimations and Lipid Profile

Serum samples from the chosen patients were allowed to defrost prior to biochemical analysis. HbA1c was analyzed directly using ion-exchange high-performance liquid chromatography (HPLC) with commercially available reagents (D-10 Hemoglobin A1c Program #12000949) in a fully automated system (D-10 Hemoglobin Testing System #12010405, Bio-Rad, Hercules, CA, USA). Fasting serum insulin levels were analyzed using a Luminex multiplex system (Luminex Corp, Austin, TX, USA) and a commercially available kit, catalog no. HINS-MAG. The intra-assay % CV was <10 and the inter-assay % CV was <15, according to the manufacturer’s protocol. Serum glucose, triglyceride, cholesterol and high-density lipoprotein (HDL) levels were analyzed regularly using commercially purchased kits (catalog nos. 981812, 981379, 981823 and 981301, respectively) and an automated biochemical analyzer (Konelab 20 Thermo Fischer, Espoo, Finland), as we described previously [23].

### 4.5. Serum NLRP3 Estimation

Serum concentration of NLRP3 was analyzed via a commercially available enzyme-linked immunosorbent assay (ELISA) kit, catalog number CSB-E15885h, Cusabio, Houston, TX, USA. Consistent with the manufacturer’s protocol, the minimum detectable dose for this assay kit was <0.04 ng/mL of human NLRP3 with % CVs of <8% and <10% for intra- and inter-assay precision, as we described previously [23].

### 4.6. Serum IL (1α, 1β, 18, 33 and 37) Estimations

Serum concentrations of IL-18, IL-1β and IL-37 were analyzed using the Flex MAP-3D System (Luminex Corporation, Austin, TX, USA), which used human cytokine magnetic bead panels (IL-18 and IL-1β: HCYTA-60K human cytokine, chemokine growth factor panel A—immunology multiplex assay) (IL-37: HCYP4MAG-64K human cytokine chemokine magnetic bead panel IV). The intra- and inter-assay % CVs for IL-18, IL-1β and IL- 37 were <15 and <20 and <10 and <15, respectively. Serum concentrations of IL-1α and IL-33 were analyzed via ELISA kits (Bio Vendor, R & D systems, Brno, Czech Republic), catalog nos. RAF045R and RAF064R, respectively. The intra-assay % CVs for both were <5.4% and 4.7%, respectively, while the inter-assay % CVs for both were <10% and 6.9%, respectively. As we described previously [23], all standards and controls applied in all the biochemical assays were frequently assessed by the quality assurance group at the CBCD lab at the College of Science at KSU, Riyadh, KSA.

### 4.7. Data Analysis

SPSS version 22.0 (IBM, Chicago, IL, USA) was used to analyze data. Continuous normal data were presented as means ± standard deviations (SDs), while non-normally distributed variables were presented as median (25th and 75th) percentiles. Categorical data were presented as frequencies and percentages (%). All continuous variables were assessed for normality using the Kolmogorov–Smirnov test. Non-normal variables were log-transformed prior to parametric analysis. Paired *t*-tests and Wilcoxon tests were performed to compare differences over time in normal and non-normal parameters, respectively. Pearson and Spearman correlation analyses of NLRP3 with other parameters were performed at baseline and the time-interval change. Boxplot and correlation figures were plotted in MS Excel. A *p* < 0.05 was considered statistically significant.

## Figures and Tables

**Table 1:** Clinical characteristics of PD participants over time according to (**A**) FSG and (**B**) HbA1c levels.

(A) FSG									
Parameters	Control			PD			T2DM		
	Baseline	Follow-Up	*p*-Value	Baseline	Follow-Up	*p*-Value	Baseline	Follow-Up	*p*-Value
N (M/F)	13 (5/8)			48 (12/36)			6 (3/3)		
Age (years)	38.5 ± 6.2			43.1 ± 7.9			39.5 ± 11.2		
Weight (kg)	80.1 ± 19.1	80.4 ± 20.1	0.9	83.0 ± 13.7	82.6 ± 14.2	0.4	84.9 ± 3.7	82.9 ± 3.9	0.4
BMI (kg/m^2^)	30.8 ± 6.4	30.9 ± 6.9	0.9	33.7 ± 5.4	33.6 ± 5.7	0.5	33.0 ± 1.8	32.1 ± 3.5	0.4
Waist (cm)	90.8 ± 11.9	90.4 ± 10.9	0.7	96.2 ± 11.8	96.2 ± 11.9	1.0	97.6 ± 19.2	100.2 ± 13.9	0.5
Hips (cm)	105.8 ± 9.6	106.7 ± 13.3	0.3	111.7 ± 9.1	110.9 ± 8.7	0.1	116.2 ± 3.3	108.0 ± 11.5	0.3
WHR	0.9 ± 0.03	0.9 ± 0.04	0.3	0.9 ± 0.1	0.9 ± 0.1	0.2	0.8 ± 0.2	0.9 ± 0.1	0.3
SBP (mmHg)	122.5 ± 10.5	118.9 ± 8.2	0.3	121.5 ± 15.7	118.7 ± 13.4	0.2	126.0 ± 11.6	131.8 ± 12.5	0.1
DBP (mmHg)	74.0 ± 10.2	71.5 ± 10.6	0.4	77.7 ± 10.9	75.7 ± 10.9	0.2	77.0 ± 16.0	74.8 ± 12.5	0.5
Glucose (mmol/L)	6.0 ± 0.3	4.6 ± 1.0	<0.01	5.9 ± 0.3	6.0 ± 0.3	0.047	6.1 ± 0.3	8.8 ± 1.2	0.002
HbA1c (%)	5.8 ± 0.3	5.8 ± 0.8	0.8	5.5 ± 0.5	5.7 ± 1.3	0.3	5.7 ± 0.4	6.5 ± 1.3	0.2
Insulin (uU/mL)	17.7 ± 5.5	18.4 ± 6.8	0.3	16.2 ± 5.1	15.6 ± 5.0	0.005	17.8 ± 2.6	17.3 ± 2.1	0.4
TC (mmol/L)	4.8 ± 0.8	4.7 ± 1.1	0.9	4.9 ± 1.0	5.0 ± 1.2	0.5	4.1 ± 0.9	4.5 ± 0.8	0.1
HDL-C (mmol/L)	1.0 ± 0.2	1.1 ± 0.32	0.1	1.1 ± 0.3	1.1 ± 0.4	0.8	1.0 ± 0.3	1.0 ± 0.3	0.9
TG (mmol/L)	1.3 (0.9–2.1)	1.3 (1.0–2.2)	0.9	1.6 (1.1–2.4)	1.6 (1.2–2.4)	0.8	1.0 (0.8–1.6)	1.4 (1.0–2.0)	0.046
**(B) HbA1c**									
N (M/F)	31 (7/24)			28 (9/19)			8 (4/4)		
Age (years)	41.7 ± 8.2			42.1 ± 7.3			41.8 ± 10.7		
Weight (kg)	81.3 ± 15.9	80.0 ± 16.4	0.02	82.0 ± 11.7	82.2 ± 122	0.9	89.6 ± 14.1	90.5 ± 15.8	0.7
BMI (kg/m^2^)	32.8 ± 6.6	32.3 ± 6.6	0.02	33.2 ± 3.2	33.3 ± 3.9	0.8	34.4 ± 6.9	34.8 ± 7.9	0.6
Waist (cm)	98.4 ± 14.7	97.4 ± 15.1	0.1	93.4 ± 7.5	94.2 ± 8.3	0.5	89.6 ± 9.1	91.9 ± 6.2	0.2
Hips (cm)	113.0 ± 12.4	111.7 ± 12.3	0.1	108.7 ± 5.3	108.7 ± 5.3	1.0	110.5 ± 6.7	107 ± 9.4	0.4
WHR	0.9 ± 0.1	0.87 ± 0.1	0.7	0.9 ± 0.1	0.87 ± 0.1	0.5	0.8 ± 0.1	0.86 ± 0.03	0.3
SBP (mmHg)	120 ± 13.9	117.4 ± 14.0	0.1	125.9 ± 16.4	121.9 ± 12.9	0.3	118.1 ± 8.4	121.0 ± 6.5	0.4
DBP (mmHg)	75.7 ± 10.9	73.6 ± 11.3	0.2	79.1 ± 12.5	76.0 ± 9.8	0.2	76.0 ± 5.7	74.3 ± 7.9	0.9
Glucose (mmol/L)	5.9 ± 0.3	5.8 ± 0.9	0.3	5.9 ± 0.3	6.1 ± 1.3	0.4	6.1 ± 0.4	6.5 ± 1.6	0.5
HbA1c (%)	5.4 ± 0.5	5.1 ± 0.5	0.02	5.8 ± 0.3	5.9 ± 0.2	0.046	5.8 ± 0.3	8.4 ± 1.7	0.006
Insulin (uU/mL)	16.0 ± 3.7	15.7 ± 4.1	0.1	16.3 ± 5.9	15.8 ± 5.8	0.004	19.1 ± 6.1	18.0 ± 7.2	0.5
TC (mmol/L)	4.8 ± 0.9	5.1 ± 1.4	0.2	4.8 ± 0.9	4.6 ± 0.8	0.4	5.1 ± 1.5	5.3 ± 1.4	0.7
HDL-C (mmol/L)	1.1 ± 0.3	1.1 ± 0.4	0.7	1.1 ± 0.2	1.0 ± 0.3	0.7	1.0 ± 0.2	1.0 ± 0.3	0.7
TG (mmol/L)	1.3 (1.0–2.0)	1.4 (1.1–2.0)	0.4	1.5 (1.1–2.6)	1.9 (1.3–2.4)	0.5	2.1 (1.3–2.2)	1.6 (1.0–2.6)	0.6

Note: Data presented as means ± SDs and median (25th–75th) percentiles for normal and non-normal parameters. BMI, body mass index; WHR, waist–hip ratio; SBP, systolic blood pressure; DBP, diastolic blood pressure; HbA1c, glycated hemoglobin; TC, total cholesterol; HDL-C, high-density lipoprotein cholesterol; TG: triglycerides. *p*-value significant at <0.05.

**Table 2:** Circulating levels of NLRP3 and related ILs (1α, 1β, 18, 33 and 37) of PD participants over time according to (**A**) FSG and (**B**) HbA1c levels.

Parameters	Control			PD			T2DM		
	Baseline	Follow-Up	*p*-Value	Baseline	Follow-Up	*p*-Value	Baseline	Follow-Up	*p*-Value
N (M/F)	13 (5/8)			48 (12/36)			6 (3/3)		
**(A) FSG**									
IL-33 (pg/mL)	3.2 (1.1–4.1)	0.7 (0.6–1.2)	0.007	3.3 (2.9–3.9)	1.0 (0.6–3.0)	<0.001	3.1 (2.8–3.9)	3.1 (0.9–3.9)	0.3
IL-37 (pg/mL)	2.3 (1.9–4.3)	2.1 (1.9–4.7)	0.50	4.2 (2.1–10.7)	2.9 (2.1–2.9)	<0.001	2.0 (1.6–4.3)	2.3 (1.6–4.5)	0.3
NLRP3 (ng/mL)	0.1 (0.1–0.1)	0.1 (0.1–0.1)	0.9	0.13 (0.1–0.2)	0.11 (0.1–0.18)	0.01	0.2 (0.1–0.3)	0.2 (0.1–0.4)	0.3
**(B) HbA1c**									
IL-1α (pg/mL)	0.8 (0.5–1.3)	1.0 (0.9–1.2)	0.2	0.7 (0.6–1.1)	1.0 (0.8–1.2)	0.07	0.6 (0.5–0.8)	1.0 (0.9–1.4)	0.046
IL-33 (pg/mL)	3.2 (0.7–4.0)	0.8 (0.6–2.1)	<0.001	3.3 (3.0–4.0)	1.0 (0.6–2.1)	<0.001	3.2 (3.1–3.8)	0.8 (0.6–1.3)	0.2

Note: Data presented as means ± SDs and median (25th–75th) percentiles for normal and non-normal parameters; *p*-value significant at <0.05.

## Data Availability

Data can be obtained upon request from the corresponding author.

## Supplementary Materials

The following supporting information can be downloaded at https://www.mdpi.com/article/10.3390/ijms241813837/s1.
