# Supplementary material for: Acute Glycemic Control in Prediabetes Individuals Favorably Alters Serum NLRP3 Inflammasome and Related Interleukins
Source: Int J Mol Sci. 2023 Sep 8;24(18):13837. doi: 10.3390/ijms241813837 (PMC10530894; doi:10.3390/ijms241813837)
Supplement: Supplementary file 1 [file ijms-24-13837-s001.zip › ijms-2575059-supplementary.pdf]

Table S1. Baseline general characteristics and circulating levels of NLRP3 inflammasome and related interleukins of the PD participants.

Parameters	
N (M/F)	67 (20/47)
Age (year)	41.9 ± 8.0
Weight (kg)	82.6 ± 14.2
BMI (kg/m^2)	33.2 ± 5.5
Waist (cm)	95.4 ± 11.9
Hips (cm)	111.0 ± 9.6
Waist Hip Ratio	0.86 ± 0.08
Systolic BP (mmHg)	122 ± 14.5
Diastolic BP (mmHg)	77.0 ± 10.9
Fasting Glucose (mmol/L)	5.95 ± 0.30
Insulin (uU/mL)	16.48 ± 5.03
HbA1c (%)	5.61 ± 0.44
Total Cholesterol (mmol/L)	4.82 ± 0.95
HDL Cholesterol (mmol/L)	1.07 ± 0.30
Triglycerides (mmol/L)	1.49 (1.0-2.2)
IL-1α (pg/ml)	0.67 (0.5-1.2)
IL-1β (pg/ml)	0.82 (0.6-2.2)
IL-18 (pg/ml)	18.49 (4-51.5)
IL-33 (pg/ml)	3.24 (2.9-3.9)
IL-37 (pg/ml)	2.98 (2.1-8.5)
Caspase-1 (ng/ml)	0.72 (0.4-2.2)
NLRP3 (ng/ml)	0.12 (0.09-0.2)

Note: Data presented Mean ± S.D and Median (25th-75th) percentile for normal and non-normal parameters.
